# Soluble B‐cell Maturation Antigen in Multiple Myeloma and Correlation With Response to Therapy

**DOI:** 10.1155/ah/6664621

**Published:** 2025-10-15

**Authors:** Souvik Saha, Prankrishna Kakati, Kulwant Singh, Manish Kumar Singh, Khaliqur Rahman, Sanjeev Yadav, Dinesh Chandra, Ruchi Gupta, Rajesh Kashyap

**Affiliations:** ^1^ Department of Hematology, Sanjay Gandhi Postgraduate Institute of Medical Sciences, Lucknow, Uttar Pradesh, India, sgpgi.ac.in

**Keywords:** B-cell maturation antigen, monitoring, multiple myeloma, therapy response

## Abstract

**Aim:**

B‐cell maturation antigen (BCMA) is a nontyrosine kinase receptor expressed during plasma cell differentiation. Binding of ligands such as APRIL and BAFF to BCMA on malignant plasma cells leads to proliferation of tumor cells and plays an important role in the pathogenesis of multiple myeloma. Recent studies have explored the role of serum‐soluble BCMA (sBCMA) in assessing tumor load as well as monitoring of response. In this study, we aimed to detect serum sBCMA levels in newly diagnosed multiple myeloma patients as well as monitor the levels at follow‐up to correlate with response to therapy.

**Methods:**

This was a prospective, longitudinal study conducted between March 2023 and July 2024. We documented the routine disease characteristics along with sBCMA levels at baseline and followed up sBCMA levels as a marker of therapy response.

**Results:**

Baseline sBCMA levels were significantly higher in patients presenting with anemia and hypercalcemia and in patients having high‐risk cytogenetics. There was a trend toward correlation of sBCMA levels with bone marrow plasma cell percentage, β2‐microglobulin, and thrombocytopenia. We also found significant association of the sBCMA level with both ISS and R‐ISS staging. Furthermore, we assessed the response to therapy in terms of IMWG response criteria and sBCMA‐based response. The response according to conventional response criteria correlated significantly with sBCMA‐based response. We also found a significant association of decline in sBCMA levels to that of M band on response to therapy. There are certain advantages of using sBCMA as a response monitoring tool, such as in patients with renal impairment and in nonsecretory myeloma.

**Conclusion:**

Our study provides an important insight into the relation of sBCMA to disease characteristics and the kinetics of decline in sBCMA on response to therapy.

## 1. Introduction

Multiple myeloma is characterized by clonal proliferation of plasma cells and accounts for 12%–15% of all hematologic neoplasms [1]. According to the IARC, the global age standardized multiple myeloma incidence rate is 1.4 per 100,000 [2]. In India, the incidence of myeloma is estimated to be 1/100,000 [3]. Although the advent of different classes of drugs such as proteasome inhibitors and immunomodulatory drugs along with monoclonal antibodies have improved survival, most of the patients will eventually develop relapse refractory disease. Hence, newer immunotherapies targeting specific antigens are being developed. B‐cell maturation antigen (BCMA) or tumor necrosis factor receptor super family (TNFRSF17) is one such selective target antigen. It is a nontyrosine kinase receptor, which is absent on naive B‐cells. It is expressed during plasma cell differentiation and helps in survival of plasmablasts and plasma cells [4]. Recent studies have explored the role of serum sBCMA in assessing tumor load as well as monitoring of response. Its rapid turnover in blood and being independent of renal function offers distinct advantages over conventional response assessment methods. In this study, we aimed to detect serum sBCMA levels in newly diagnosed multiple myeloma patients as well as monitor the levels at follow‐up to correlate with response to therapy.

## 2. Materials and Methods

This was a prospective, longitudinal study conducted between March 2023 and July 2024 at the Department of Hematology, SGPGIMS. We included all newly diagnosed patients of multiple myeloma presenting at our institute. Relapse/refractory multiple myeloma, monoclonal gammopathy of undetermined significance (MGUS), monocloncal gammopathy of renal significance (MGRS) and smoldering multiple myeloma (SMM) were excluded from the study. The objectives were (1) to study the relationship of sBCMA levels to baseline disease characteristics and (2) correlation of sBCMA levels with response to therapy.

Serum for sBCMA was collected from 85 newly diagnosed patients along with routine baseline investigations, including bone marrow examination and fluorescent in situ hybridization (FISH), serum protein electrophoresis, serum immunofixation, serum free light chain assay, and features of end organ damage. ISS and R‐ISS staging was done for all patients. We also included 4 healthy control subjects.

### 2.1. Sandwich ELISA

Assessment of serum sBCMA was done using enzyme‐linked immunosorbent assay with anti‐BCMA antibody (Human BCMA/TNFRSF 17 DuoSet ELISA DY193—R&D Systems, Minneapolis, USA). Frozen serum samples were thawed and initially tested at a 1:500 dilution. The samples with very high values were retested at 1:1000 and 1:2000 dilutions. The data were recorded with ELISA plate reader (Multimode Microplate Reader, BioTek Synergy H1), and the quantitative analysis of samples was carried out using a four parameter logistic (4PL) curve fit available with Gen 5 software.

### 2.2. Follow‐Up

The patients were assessed at 4 months, 8 months, and 1 year of initiation of therapy. The evaluation included myeloma panel along with serum sBCMA levels. Bone marrow examination was not done at follow‐up. The patients were classified as very good partial response (VGPR), partial response (PR), stable disease (SD), and progressive disease (PD) according to IMWG response criteria. At follow‐up, response in terms of BCMA levels was also assessed according to the previously published literature [5]. VGPR was defined as > 90% reduction in sBCMA levels, PR was defined as 50%–90% reduction in sBCMA levels, and SD was defined as < 50% reduction in sBCMA levels as compared to baseline. We aimed to correlate sBCMA‐based response with standard response criteria in our study.

Statistical analysis was done using IBM SPSS 25. Baseline sBCMA was non‐normally distributed. Mean, median, and interquartile range were used for descriptive statistics. Mann–Whitney U test was applied to test the association of sBCMA with sex, plasma cell leukemia, anemia, hypercalcemia, renal injury, and cytogenetics. Kruskal–Wallis H test was used for ISS and R‐ISS staging. Spearman rank correlation was used to test linear relationship between sBCMA and quantitative variables including age, total leukocyte count (TLC), platelet count, plasma cell percentage in bone marrow, serum albumin, lactate dehydrogenase (LDH), and β2‐microglobulin.

## 3. Results

We included a total of 85 patients in the study. 12 patients were lost to follow‐up after diagnosis. Two patients died at diagnosis and 2 more patients died during the course of therapy. We could follow up 7 patients till 1 year, 17 patients till 8 months, and 23 patients till 4 months until the end of the study period. We could evaluate only baseline characteristics in 23 patients.

The median age of study population was 58 years (range 31–80 years). The male:female ratio was 2.5:1. Mean hemoglobin at diagnosis was 8 g/dL. 72 patients (84.7%) presented with anemia, 35 patients (41.2%) presented with renal injury, and 12 patients (14.1%) presented with hypercalcemia at baseline. The median plasma cell percentage in bone marrow was 50%. On immunofixation, the commonest type was IgG kappa (30.6%), followed by IgG lambda (24.7%) and kappa LC (21.2%). On FISH analysis, 53% patients did not have any cytogenetic abnormalities. The commonest cytogenetic abnormality was gain 1q21 in 23% patients followed by t(4; 14) in 10% patients. The different cytogenetic abnormalities are depicted in Supporting Information Figure 1. Thirteen patients had more than one cytogenetic abnormality. According to ISS staging, majority of patients belonged to ISS Stage III (76.5%) while according to R‐ISS staging, majority of patients belonged to R‐ISS Stage II (56.5%). The baseline characteristics are listed in Table 1.

**Table 1:** Baseline characteristics of the study population.

	Total (*n* = 85)
Median age in years (range)	58 (31–80 years)
Sex	
Male	61 (71.8%)
Female	24 (28.2%)
Anemia	72 (84.7%)
Renal injury	35 (41.2%)
Hypercalcemia	12 (14.1%)
Plasma cell leukemia	3 (3.5%)
Median TLC	5830/dL
Median platelet count	150 × 10^9^/L
Median bone marrow plasma cells	50%
Immunofixation	
IgG kappa	26 (30.6%)
IgG lambda	21 (24.7%)
IgA kappa	6 (7.1%)
IgA lambda	3 (3.5%)
Kappa LC	18 (21.2%)
Lambda LC	11 (12.9%)
Cytogenetics	
High risk	30 (35.3%)
Standard risk	55 (64.7%)
Median beta 2 microglobulin (range)	7.5 (2.8–46.9)
ISS staging	
Stage I	1 (1.2%)
Stage II	19 (22.4%)
Stage III	65 (76.4%)
R ISS staging	
Stage I	1 (1.2%)
Stage II	48 (56.5%)
Stage III	36 (42.3%)

The median sBCMA level in healthy controls was 36.5 ng/mL. sBCMA levels in multiple myeloma patients, at baseline, ranged from 57.1 to 3529.8 ng/mL with a median level of 298.4 ng/mL. Hence, compared to healthy controls, sBCMA levels were much higher in myeloma patients.

There were no differences in sBCMA levels according to age and sex of the patients. The three patients with plasma cell leukemia had higher median sBCMA level, but was not statistically significant. Patients presenting with anemia had a significantly higher median sBCMA level (327.9 ng/mL) as compared to patients without anemia (182 ng/mL) (*p* = 0.018 by Mann–Whitney test). Similarly, patients with hypercalcemia had a higher median sBCMA level (574.6 vs. 289.4 ng/mL), which achieved statistical significance (*p* = 0.049 by Mann–Whitney test). Patients presenting with renal injury also had a higher median sBCMA level (362.5 vs. 292.2 ng/mL); however, the difference did not reach statistical significance (*p* = 0.131 by Mann–Whitney test). When grouped according to cytogenetics, 55 patients had standard risk (SR), while 30 patients had high risk (HR) cytogenetics. Median sBCMA level was significantly higher in the HR group (684.4 ng/mL) as compared to the SR group (234.9 ng/mL) (*p* < 0.01 by Mann–Whitney test). Table 2 demonstrates the comparison of sBCMA according to the disease characteristics.

**Table 2:** Correlation of baseline sBCMA levels with disease characteristics.

Parameters	Median baseline sBCMA (ng/mL)		*p* value
	Yes	No	
Anemia	327.9 (57.1–3529.8)	182 (88.2–914.3)	0.018
Renal injury	362.5 (88.4–3529.8)	292.2 (57.1–1018.1)	0.131
Hypercalcemia	574.6 (161.4–987.4)	289.4 (57.1–3529.8)	0.049
Plasma cell leukemia	659.8 (327.8–966.8)	292.2 (57.1–3529.8)	0.134
High risk cytogenetics	684.4 (120.4–3529.8)	234.9 (57.1–914.3)	< 0.01

**Figure 1:**
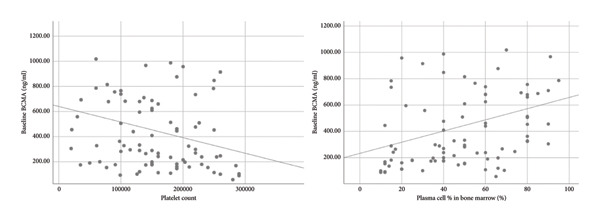
Baseline sBCMA showed weak negative correlation with platelet count and moderate positive correlation with bone marrow plasma cells.

**Figure 2:**
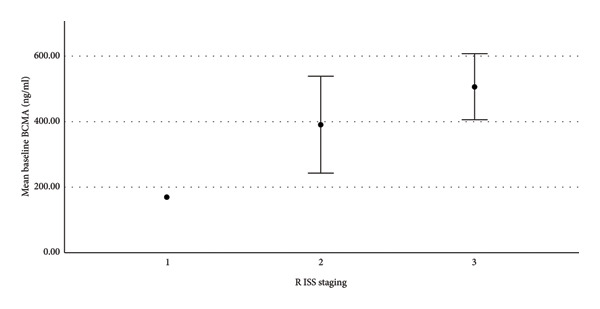
sBCMA levels significantly correlated with R ISS staging (*p* = 0.01).

There was a moderate strength of correlation between sBCMA and bone marrow plasma cell percentage (correlation coefficient 0.406) (Figure 1). We also found weak correlation with β2‐microglobulin (correlation coefficient 0.351) and thrombocytopenia (correlation coefficient 0.301) (Figure 1). Supporting Information Figure 2 shows the relationship of baseline sBCMA with β2‐microglobulin. According to ISS staging, mean sBCMA levels in Stage III was 493.5 ng/mL, in Stage II was 258.9 ng/mL, and in Stage I was 168.7 ng/mL. Supporting Information Figure 3 shows the significant correlation between sBCMA and ISS staging. Similarly, according to R‐ISS staging, mean sBCMA levels in Stage III was 506.8 ng/mL, Stage II was 390.7 ng/mL ,and Stage I was 168.7 ng/mL (Figure 2). Hence, there was a significant correlation between sBCMA levels and both ISS (*p* = 0.01 by Kruskal–Wallis test) and R‐ISS staging (*p* = 0.01 by Kruskal–Wallis test). There was no correlation with TLC, serum albumin, and LDH levels. Supporting Information Figures 4 and 5 show the relationship of baseline sBCMA with TLC and LDH levels, respectively.

Furthermore, we included all significant variables in the generalized linear model for identification of independent predictors of sBCMA. A total of 8 independent variables (i.e., anemia, hypercalcemia, cytogenetics, platelet count, bone marrow plasma cell percentage, β2‐microglobulin, and ISS and R‐ISS staging) were included in the model, those that were statistically significant in univariate analysis. In the final model, cytogenetics, β2‐microglobulin, and thrombocytopenia were found to be independent predictors for sBCMA.

**Figure 3:**
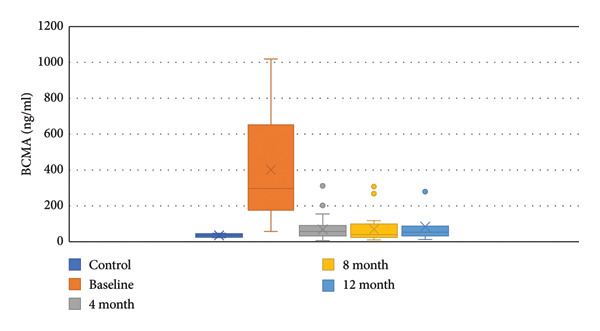
sBCMA levels at baseline and at follow‐up.

At follow‐up, responses were evaluated according to conventional IMWG criteria and sBCMA‐based criteria at 4, 8, and 12 months since diagnosis. According to standard response criteria, at 4 months, VGPR was achieved in 48%, PR in 48%, and SD in 4% patients. At 8 months, 58.4% achieved VGPR, 33.3% achieved PR, and 8.3% had SD. At 12 months, 57% patients were in VGPR and 43% were in PR. According to sBCMA‐based criteria, at 4 months, VGPR was achieved in 35.4%, PR in 52.1%, and SD in 12.5% patients. At 8 months, 54.2% achieved VGPR, 29.2% achieved PR, and 16.6% had SD. At 12 months, 42.9% patients had VGPR, 42.9% had PR, and 14.2% had SD. Figure 3 shows decline in sBCMA levels in response to therapy. There was a significant correlation between the conventional response and sBCMA‐based response at 4, 8, and 12 months of therapy (*p* < 0.05 by Chi square test). Supporting Information tables 1, 2, and 3 show the correlation of sBCMA‐based response with conventional response at 4, 8, and 12 months, respectively. We did not find any significant differences in sBCMA levels according to survival status of the patients.

We also tried to find the relationship of decline in sBCMA levels with decline in involved free light chain (iFLC), serum free light chain ratio (SFLC), and M band on response to therapy. Table 3 shows the mean sBCMA, iFLC, SFLC, and M band and percentage decrease in their levels at different time points. Figure 4 also shows the decline in their levels on response to therapy. There was a significant correlation of decline in sBCMA with that of M band upto 4 months of therapy (*p* = 0.001 by paired T test). However, there was no correlation with iFLC (*p* = 0.1 by paired T test) and SFLC (*p* = 0.2 by paired T test). Since the number of patients followed up beyond 4 months was few, we could not find any statistical correlation beyond 4 months.

**Table 3:** Mean sBCMA, iFLC, SFLC, and M band at different timepoints with percentage decline from baseline.

	Base line	4 months	8 months	12 months
sBCMA (ng/mL)	424.59	84.46 (80.1%)	76.66 (81.9%)	84.19 (80.1%)
iFLC	2836	244.33 (91.3%)	105.8 (96.2%)	55.54 (98%)
SFLC	92.99	11.74 (87.3%)	4.5 (95.1%)	2.09 (97.7%)
M Band (g/dL)	2.61	0.44 (83.1%)	0.24 (90.8%)	0.34 (86.9%)

Figure 4(a–d) Decline in mean sBCMA, iFLC, SFLC, and M band at different timepoints on response to therapy.

**Figure:**
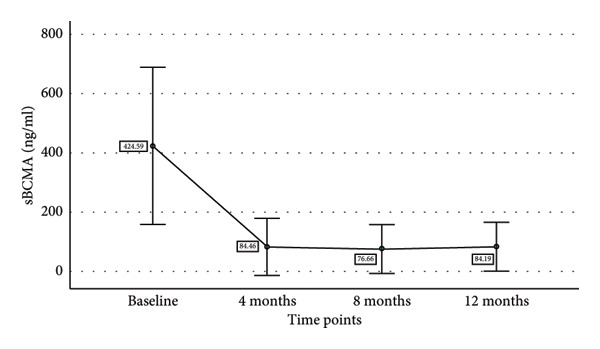
(a)

**Figure:**
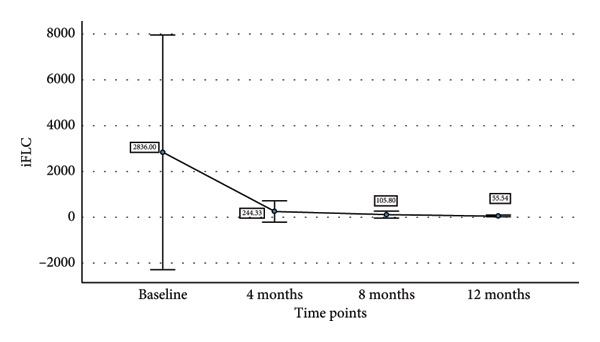
(b)

**Figure:**
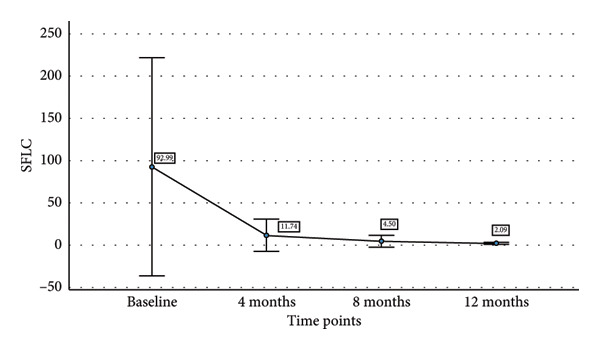
(c)

**Figure:**
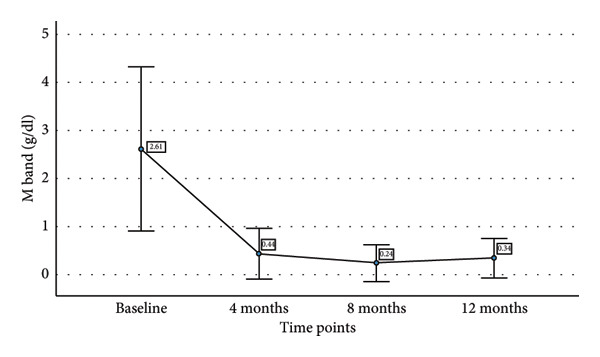
(d)

## 4. Discussion

BCMA is a TNFR superfamily member expressed almost exclusively on plasma cells [5]. It has 2 ligands: A proliferation‐induced ligand (APRIL) and B‐cell activating factor (BAFF). Binding of these ligands to BCMA on malignant plasma cells lead to proliferation of tumor cells [6]. There are various mechanisms involved like activation of nuclear factor kappa B pathway and upregulation of antiapoptotic proteins [6, 7]. APRIL is more important for plasma cell proliferation because of 2 reasons—(a) it has a stronger affinity for BCMA [7] and (b) it binds to TACI (transmembrane activator and calcium modulator). TACI, highly expressed on multiple myeloma cells is associated with increased survival of malignant plasma cells [4]. Low expression of BCMA is also found on plasmacytoid dendritic cells, which aids in tumor progression [8]. BCMA on plasma cells is acted upon by gamma secretase, releasing soluble BCMA fragment (sBCMA) into serum [7].

A number of BCMA targeted therapies have been introduced for relapse refractory multiple myeloma including—(1) CART therapy (Idecabtagene vicleucel and Ciltacabtagene autoleucel), (2) antibody drug conjugate (Belantamab mafodotin), and (3) bispecific antibodies (Teclistamab) [9]. Measurement of sBCMA was introduced to assess response to BCMA‐targeted CART therapy. Responding patients have a significant decrease in sBCMA levels as compared to nonresponders. Similarly, increasing sBCMA levels indicate loss of efficacy of CART therapy [10].

Measurement of sBCMA levels found its use in other settings as well. A number of studies have already shown significantly increased sBCMA levels in multiple myeloma patients [5, 11, 12]. Furthermore, MGUS and SMM patients with significantly higher baseline sBCMA levels have increased progression rates to multiple myeloma [13]. It has also been shown to be a valuable marker for the prognosis and treatment response [6, 7, 11].

Currently, measurement of M protein and SFLC are used to monitor response to therapy. However, there are a few disadvantages associated with such assessment. Firstly, SFLC is falsely elevated in patients with renal injury as light chains are excreted by the kidney [14]. This is significant since renal impairment occurs in more than 20% of myeloma patients and renal reversibility occurs in less than 50% of patients [15]. On the other hand, sBCMA is not affected by renal function and can be used to monitor response independent of renal function [5]. Secondly, in nonsecretory and oligo‐secretory myeloma, response assessment has to be done by bone marrow examination and PET CT, which are invasive, inconvenient, and expensive modalities [16]. Hence, patients are not properly followed up and often presents with a clinical relapse. sBCMA levels have been shown to be an effective response assessment modality in such patients [11]. Thirdly, M protein has a long half‐life, while sBCMA has a very short half‐life of 24–36 h [17]. This allows for rapid changes in levels in response to tumor burden. All of these factors have encouraged studies to evaluate the role of sBCMA levels as a response assessment tool.

In our study, we aimed to look for the association of baseline sBCMA levels with the disease characteristics. Furthermore, we aimed to correlate sBCMA‐based response with standard IMWG response criteria as a marker of response to therapy.

We found significantly higher sBCMA levels in multiple myeloma patients as compared to healthy controls. This has already been established in various studies, which had shown a progressively increasing trend of sBCMA level in MGUS, SMM, and multiple myeloma patients, when compared to normal individuals [5, 11, 12]. We found significantly increased levels of baseline sBCMA in patients presenting with anemia and hypercalcemia. There was also a trend of increased sBCMA in patients with renal injury; however, it was not statistically significant. None of the earlier studies had commented on such correlation of sBCMA levels with end organ damage.

Sanchez et al. had published one of the initial studies on sBCMA level measurement, and they reported that BCMA levels correlated with bone marrow plasma cells [6]. This finding was widely validated in later studies [5, 11, 18]. However, we found only a moderate strength of correlation as opposed to strong correlation in the previously published literature. We observed a weak correlation between sBCMA and β2‐microglobulin. This was in accordance to the previously published literature [5, 18]. There was also a weak correlation between sBCMA and thrombocytopenia. Such an association has not been reported in any study till date. We found significantly higher sBCMA levels in patients with HR cytogenetics as compared to SR cytogenetics. Contrary to our findings, Elmeliegy et al. in their study did not find any statistically significant difference between HR and SR cytogenetics [18]. In our study, there was statistically significant correlation with both ISS and R‐ISS staging systems. Similar to our study, sBCMA was significantly associated with ISS and R‐ ISS staging in a report published in 2023 [18]. On multivariate analysis, we found cytogenetics, β2‐microglobulin, and thrombocytopenia to be independent predictors of sBCMA levels.

We followed up the patients at 4, 8, and 12 months of therapy. At each timepoint, we assessed the response according to IMWG response criteria and sBCMA response criteria. We found a significant correlation of the sBCMA‐based response with the conventional response. There was also a significant correlation between the decline in sBCMA levels with the decline in M band at 4 months of therapy. However, we did not find a correlation of sBCMA decline with the decrease in iFLC and SFLC. A study published by Wiedemann et al. [5] assessed sBCMA levels as early as 1 week of therapy followed by every 4 weeks. They had also observed a significant correlation with changes in M protein and SFLC at the same timepoints. Furthermore, they reported early sBCMA response at 4 weeks of therapy predicted significantly improved PFS. Thus, our findings partially corroborate with this study.

sBCMA has been evaluated as a prognostic marker in a number of publications. Ghermezi et al. showed that higher sBCMA levels correlated with poorer PFS as well as OS [11]. Similar results were observed by Sanchez et al. [6] and Guo et al. [12]. However, we did not find any correlation between baseline sBCMA levels and survival.

To the best of our knowledge, there is only one Indian study that showed higher expression of BCMA (CD 269) on malignant plasma cells by flow cytometry. They did not find any correlation of CD 269 expression with age, sex, cytogenetics, and response to therapy [19].

The major limitation of our study was the short study period. We could not assess for sBCMA response in the whole cohort till 12 months of therapy because of the short duration of study. Also, our correlation with survival was not significant because of the limited study period and small sample size. Follow‐up studies involving more number of patients with a longer study period is required. A longer study period will also allow for evaluation of sBCMA levels at relapse.

## 5. Conclusion

The sBCMA level has been studied recently as a newer monitoring and prognostic tool in multiple myeloma patients. However, it has not yet been established in the standard guidelines. In our study, we showed that sBCMA levels were higher in multiple myeloma patients as compared to healthy controls and that baseline sBCMA levels were significantly correlated with anemia, hypercalcemia, HR cytogenetics, thrombocytopenia, β2‐microglobulin, bone marrow plasma cells, and ISS as well as R‐ISS staging. We further showed that sBCMA‐based response with therapy significantly correlated with conventional response criteria. There is paucity of data on the use of sBCMA level measurement in Indian settings, and this study provides an insight into this subject.

## Ethics Statement

The study was approved by the Institution Ethics Committee vide PGI/BE/211/2023. Informed consent was obtained from all study participants.

## Conflicts of Interest

The authors declare no conflicts of interest.

## Author Contributions

Souvik Saha: conceptualization, methodology, data curation, analysis, and original draft preparation. Prankrishna Kakati and Kulwant Singh: methodology and testing of samples. Rajesh Kashyap: conceptualization, analysis, and review and editing of draft. Manish Kumar Singh, Khaliqur Rahman, Sanjeev Yadav, Dinesh Chandra, and Ruchi Gupta: review of final draft.

## Funding

This work was supported under SGPGI Intramural Grant (A‐11‐PGI/IMP/88/2023).

## Supporting Information

Figure 1: Division of patients according to cytogenetics

Figure 2: Baseline sBCMA levels showed weak positive correlation with β2‐microglobulin (*p* < 0.05).

Figure 3: Significant correlation between sBCMA and ISS staging (*p* = 0.01).

Figure 4: No correlation between sBCMA and TLC (*p* = 0.526).

Figure 5: No correlation between sBCMA and LDH (*p* = 0.3).

Table 1: Significant correlation between conventional and sBCMA‐based response at 4 months.

Table 2: Significant correlation between conventional response and sBCMA‐based response at 8 months.

Table 3: Significant correlation between conventional response and sBCMA‐based response at 12 months.

## Supporting information


**Supporting Information** Additional supporting information can be found online in the Supporting Information section.

## Data Availability

The data that support the findings of this study are available in the Supporting Information of this article.
