# Supplementary material for: Soluble B‐cell Maturation Antigen in Multiple Myeloma and Correlation With Response to Therapy
Source: Adv Hematol. 2025 Oct 15;2025:6664621. doi: 10.1155/ah/6664621 (PMC12539665; doi:10.1155/ah/6664621)
Supplement: Supplementary file 1 — Supporting Information Additional supporting information can be found online in the Supporting Information section. [file AH-2025-6664621-s001.docx]

**Soluble B cell maturation antigen in multiple myeloma and correlation with response to therapy**

Souvik Saha, Prankrishna Kakati, Kulwant Singh, Khaliqur Rahman, Manish Kumar Singh, Sanjeev Yadav, Dinesh Chandra, Ruchi Gupta, Rajesh Kashyap

Figure 1: Division of patients according to cytogenetics


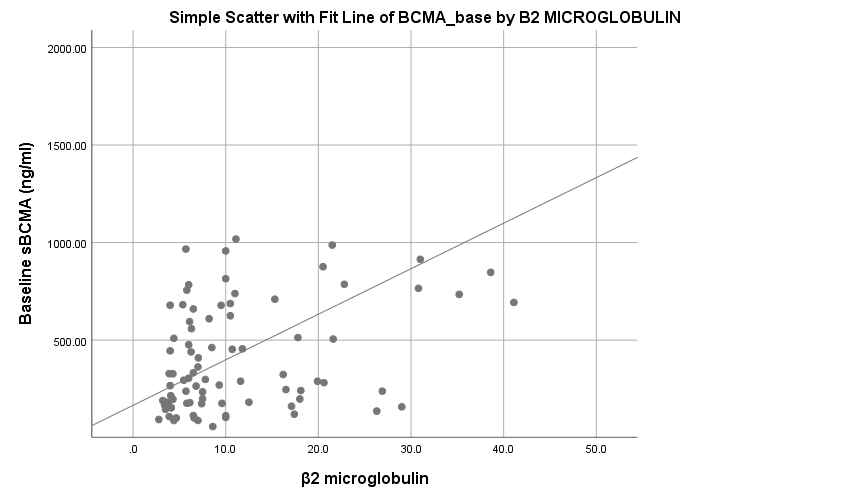


Figure 2: Baseline sBCMA levels showed weak positive correlation with β2‑microglobulin (p<0.05)


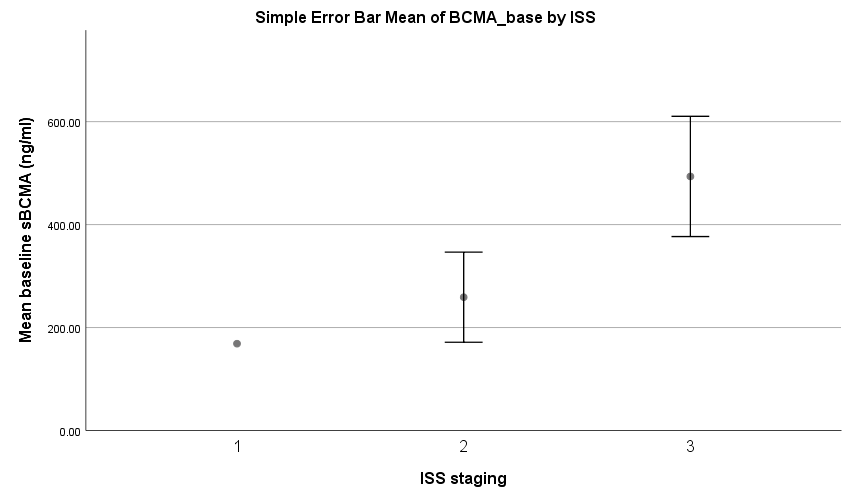


Figure 3: Significant correlation between sBCMA and ISS staging (p=0.01)


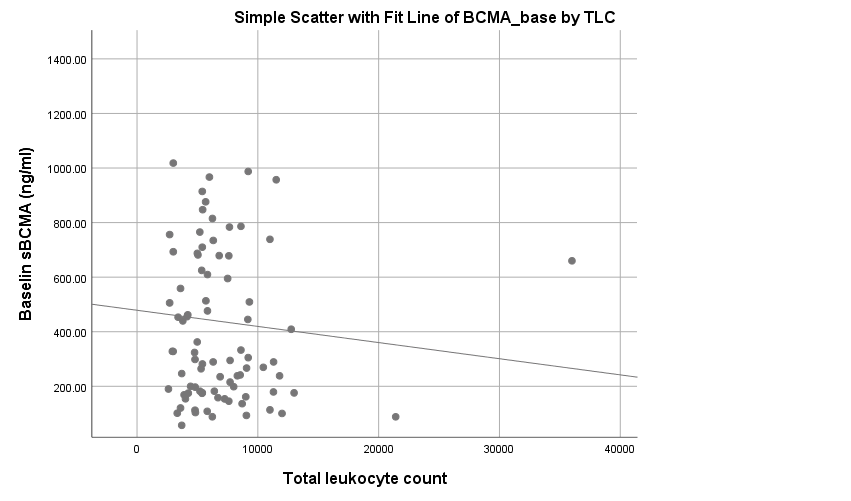


Figure 4: No correlation between sBCMA and TLC (p=0.526)


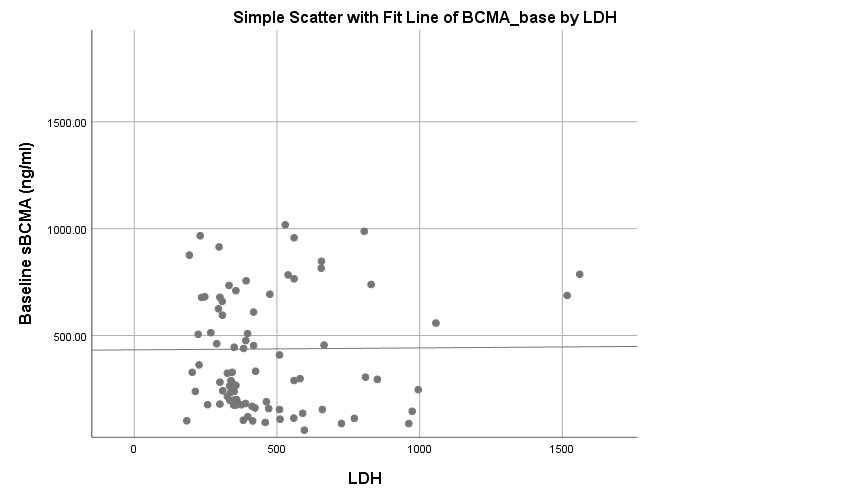


Figure 5: No correlation between sBCMA and LDH (p= 0.3)

| **Conventional Response at 4 months** | **sBCMA based response at 4 months** | | | |
| --- | --- | --- | --- | --- |
|  |  | VGPR | PR | SD |
|  | VGPR | 15 | 7 | 1 |
|  | PR | 1 | 18 | 4 |
|  | SD | 1 | 0 | 1 |

Table 1: Significant correlation between conventional and sBCMA based response at 4 months

| **Conventional response at 8 months** | **sBCMA based response at 8 months** | | | |
| --- | --- | --- | --- | --- |
|  |  | VGPR | PR | SD |
|  | VGPR | 12 | 2 | 0 |
|  | PR | 0 | 5 | 3 |
|  | SD | 1 | 0 | 1 |

Table 2: Significant correlation between conventional response and sBCMA based response at 8 months

| **Conventional response at 12 months** | **sBCMA based response at 12 months** | | | |
| --- | --- | --- | --- | --- |
|  |  | VGPR | PR | SD |
|  | VGPR | 3 | 1 | 0 |
|  | PR | 0 | 2 | 1 |

Table 3: Significant correlation between conventional response and sBCMA based response at 12 months
